# Patients seeking occupational health advice before and after COVID-19

**DOI:** 10.1093/occmed/kqag048

**Published:** 2026-06-09

**Authors:** Hadia Hazoor, Nick Pearson, Francis Creed

**Affiliations:** Sheffield Institute of Social Sciences, Sheffield Hallam University, Sheffield, S10 2BP, UK; Sheffield Occupational Health Advisory Service (SOHAS) 33, Rockingham Lane, Sheffield, S1 4FW, UK; Emeritus Professor, University of Manchester and Trustee of Sheffield Occupational Health Advisory Service, UK

## Abstract

**Background:**

The COVID-19 pandemic led to an increased prevalence of psychological disorder in population-based studies, but the effect on the ability to work has been less well studied, except in health workers.

**Aims:**

This study aimed to assess whether there has been an increase in the number of people seeking assistance for psychological problems from the Sheffield Occupational Health Advisory service in the UK since the COVID-19 pandemic.

**Methods:**

The study used routinely collected data at Sheffield Occupational Health Advisory Service (SOHAS) to compare the proportion of all new referrals presenting with psychological problems for 3 years prior to (2017–2019) and following the pandemic (2022–2024). Statistical differences were assessed using the chi-square test.

**Results:**

Psychological problems were the most common health problem presenting among those seeking advice at SOHAS both before (57.9%) and after the pandemic (61.5%); this was a small, but significant, difference (*P* < 0.01). In the post-COVID period, phone consultations largely replaced face-to-face appointments, and the patients were slightly younger, less likely to be referred by a GP and less likely to be off work through illness. The advice given post-COVID was more likely to include employment law and disability discrimination.

**Conclusions:**

The observed increase in people seeking advice with psychological problems was minor and bore no resemblance to the reported increase in mental health problems in the UK population since the COVID-19 pandemic. There was no evidence that GPs are detecting and referring more patients with psychological problems affecting work performance.

## Teaser

Over half of UK workers do not have access to occupational health services. In Sheffield, anyone can seek advice about health problems interfering with work at the Sheffield Occupational Health Advisory Service. This project assessed whether the proportion seeking advice for mental health problems increased after the COVID-19 pandemic, as reported in the general population. Psychological problems were the most common reason for seeking advice both before and after the pandemic.

## Introduction

There is great concern in the UK about the number of people off work because of long-term sickness; the number has almost doubled in the years 2020–2022. The largest relative increase in recent years has been among those aged 25–34 years old, and the commonest cause of long-term sickness is mental illness, phobias and nervous disorders (24% increase) [1]. It has been calculated that people aged 16–34 years who have mental health conditions are 4.7 times more likely to be economically inactive than their cohort [2].

The problem is not confined to UK, and there have been concerns globally about young people off work with depression and anxiety disorders since the COVID-19 pandemic [3–5]. The increase in mental health problems has been reported in many occupational groups, but was especially high in health and social care workers [6, 7]. There have been several calls for more research on the effect of the pandemic on employment patterns, work arrangements, worker well-being and mental health [4, 5, 8].

This study aimed to assess whether the Sheffield Occupational Health Advisory service experienced an increase in the number of people seeking assistance for psychological problems after the pandemic. The study used routinely collected data at the Sheffield Occupational Health Advisory Service (SOHAS). It assessed whether there was an increase in new referrals to the service of people with a primary complaint of psychological disorders during 3 years after the pandemic compared to 3 years prior to the pandemic.

## Methods

Sheffield Occupational Health Advisory Service (SOHAS) is a charity provider which has operated for 45 years, delivering free advice to help people with health conditions remain in work, with a particular focus on early intervention. It is different from the majority of occupational health services in UK, as it is not linked to particular workplaces and is independent of employment-based occupational health services, though SOHAS staff may liaise with such services as necessary. Patients are referred to SOHAS by their GP, or they self-refer, independent of their employer, and the SOHAS adviser provides confidential advice to the patient. The adviser may simply suggest how the patient manages the situation but may also liaise with the patient’s employer/manager when this is deemed necessary or appropriate. During the period of this study, there were 5 SOHAS advisers who worked across 22 general practices in the areas of Sheffield with the greatest socioeconomic deprivation and a higher-than-average number of people claiming work-related benefits. The work of SOHAS is focussed on the most deprived areas of Sheffield; most of these areas have an index of multiple deprivation in the range of that for the most deprived 5% of England. In Sheffield, 13.3% businesses are small or medium-sized compared to 10.4% nationally. Three quarters of referrals come from primary care with 10–15% self-referrals; the remainder are referred by secondary services such as musculoskeletal, physiotherapy, Long COVID Rehabilitation Hub or psychological treatment clinics. SOHAS sees around 800–1000 new referrals per year: most of these are in work, but half of these were on sickness absence at referral. SOHAS advises patients on interactions between health and work performance, employment law, guidance on return to work, disability discrimination and health and safety law. It is not a therapeutic agency, but the advisers can signpost the local talking therapy service, or other local support services.

At the first appointment details are recorded on a standard proforma regarding socio-demographic and health problems, plus the type of advice which is offered. In one or more sessions, patients receive advice on how to cope with their employment situation. There is no record of the availability of employer-commissioned occupational health services, but these services will only be available to a minority of patients seen at SOHAS.

For the purposes of this study, two groups were created and compared. The first, “pre-COVID” group, included attendees first seen between 1 January 2017 to 31 December 31 2019. The “post-COVID” group were seen between 1 January 2022 and 31 December 2024. We did not include data from 2020 and 2021 because the nature of the service changed considerably during that time. During the COVID-19 pandemic, there were fewer referrals overall, but there were new referrals for COVID-related issues, e.g. clinically vulnerable people expected to work on site rather than work at home, or questions over COVID-safety in workplaces.

The main analysis compared the proportion of new referrals with a psychological problem as the primary reason for referral. The two groups were also compared with respect to demographic factors, health problems, and advice received. Statistical significance was assessed using the chi-square test with collapsed groups to avoid small number is some cells.

During the pandemic, most advisers ceased visiting health centres to see patients; there was a large increase in the proportion of consultations that took place by phone instead. Whether the pattern of advice offered to patients following a telephone consultation differed from a face-to-face one was assessed for patients who were seen by all advisers, combining the pre- and post-COVID groups. Nearly all patients were seen within 2 weeks of referral, and this was so before and after COVID. There was little difference in the duration of telephone consultations and face-to-face ones; both lasted approximately 45 minutes.

All participants gave consent for the routine collected anonymised data to be used in research analyses. The project received ethical approval from the Hallam University Research Ethics Committee (ref HAZO_WITH_2025).

## Results

The numbers of participants are shown in Table 1. Fewer patients were seen in the post-COVID period compared to before the pandemic because advisers were available for fewer hours each week.

**Table 1:** Socio-demographic and referral data comparing pre- and post-COVID groups

	Pre-COVID group	Post-COVID group	
	*n* = 3555	*n* = 2183	
**Sex**			
Males	1369 (39.%)	791 (36%)	ns
Females	2186 (61%)	1392 (64%)	
**Age**			
Up to 24	205 (6%)	117 (5%)	
25–44	1257 (35%)	957 (44%)	*P* < 0.001.
45–64	1818 (51%)	1039 (48%)	
Over 65	275 (8%)	70 (3%)	
**Ethnicity**			
White British/European	3176 (89%)	1848 (85%)	
Other	379 (11%)	335 (15%)	*P* < 0.001
**Mode of referral**			
GP referral	2309 (65%)	1130 (52%)	
Self-referral	232 (7%)	275 (13%)	
Other agencies^a^	1014 (28%)	778 (35%)	*P* < 0.001
**Employment status**	(*n* = 3163)	(*n* = 2061)	
Employed	1252 (40%)	1037 (50%)	
Employed but off Sick	1632 (52%)	909 (44%)	
Unemployed	116 (3%)	101 (5%)	*P* < 0.001
Unemployed though ill health	163 (5%)	14 (1%)	

a Secondary health services such as musculoskeletal, physiotherapy, Long COVID Rehabilitation Hub or psychological treatment clinics.

The greatest difference between pre- and post-COVID groups was the nature of the first appointment. In the pre-COVID period, there were 2666 (75%) face-to-face appointments and 889 (25%) phone calls. In the post-COVID period, there were 568 (26%) face-to-face appointments and 1615 (74%) phone/video calls.

Table 1 shows there was no difference between the pre- and post-COVID groups in terms of sex distribution, but the post-COVID group included slightly more people in younger age groups (24–44 years), and slightly more from non-white ethnic groups. Self-referral was more frequent in the post-COVID group, with fewer being referred by GPs. In the post-COVID group, a greater proportion were employed, but not off sick and fewer were unemployed through illness, showing that the ethos of early intervention was maintained post-COVID.

Data for presenting health problems were limited to those patients in whom this was recorded. Some patients sought advice, but it turned out that a health problem was not the primary reason for difficulties at work. Work-related stress was usually the most common reason underlying the referral and would be recorded as “psychological problem.” A health problem (anxiety or depressive disorder and/or an additional physical illness) was often secondary rather than the primary reason for referral; when this occurred, a second health problem was recorded. The proportion with psychological problems as the main health problem was slightly increased in the post-COVID compared to the pre-COVID group (61.5% versus 57.9%), and the proportion with musculo-skeletal problems was slightly less (Table 2). When the presenting health problems were analysed as 3 groups in order to avoid cells with small numbers (psychological, musculo-skeletal and other groups), the difference between pre- and post- COVID groups was significant (chi-square = 7.32, *P* < 0.01).

**Table 2:** Health problems (no. and %) comparing pre- and post-COVID groups

	Pre-COVID group *n* = 1633	Post-COVID group *n* = 1758
	No.	No.
Chest/asthma	76 (5%)	38 (2%)
Digestive	18 (1%)	56 (3%)
Eyes, ENT, hearing	132 (8%)	49 (3%)
Reproductive	35 (2%)	43 (2%)
Heart/circulation	25 (2%)	52 (3%)
Injuries	16 (1%)	24 (2%)
Muscles/joints/RSI/back	259 (16%)	226 (13%)
Nervous system	39 (2%)	78 (4%)
Skin	12 (1%)	24 (1%)
Psychological	946 (58%)	1,082 (62%)
Problems in other systems	75 (4%)	86 (5%)

Chi-square test. Comparison of three groups (Psychological, Musculo-skeletal and other health problems^a^) chi square = 7.32, *P* < 0.01.

ENT, Ear, Nose and Throat; RSI, Repetitive Strain Injury.

a The “other health problems” group (i.e. all those except psychological and musculoskeletal): pre-COVID, *n* = 428 (26%) and post-COVID groups, *n* = 450 (26%).

The overall pattern of the advice given was similar in the pre- and post-COVID periods (Table 3). The main difference was an increase in advice being given concerning disability discrimination and employment law (aspects of which were relevant to the patients’ situation), with the corresponding reduction of advice concerning preventative advice (how to prevent the health problem worsening at work) and advice concerning how to manage the return to work.

**Table 3:** Forms of advice comparing pre- and post-COVID periods

	Pre-COVID	Post-COVID
	No = 15 250	No = 7149
**Category of advice offered**		
Preventative advice	5469 (36%)	2001 (28%)
Return to work	2692 (18%)	860 (12%)
Employment law	2804 (18%)	1698 (23%)
Disability discrimination	1191 (8%)	998 (14%)
Health and Safety, common law and other legal	184 (1%)	184 (2%)
Health service	88 (1%)	62 (1%)
Vocational guidance	338 (2%)	305 (4%)
Retirement/pension	148 (1%)	117 (2%)
Representation at appeal	99 (1%)	18 (1%)
Other	36 (1%)	7 (1%)
None	2201 (13%)	899 (12%)

Multiple categories of advice are often recorded at the initial session.

The reduction in preventative advice post-COVID cannot be explained by the increased use of phone appointments, as this advice is offered with similar frequency at phone appointments compared to face-to-face ones (Table 4). It is possible that other changes post-COVID (decrease in return to work advice and increase in advice relating to employment law and disability discrimination) might be accounted for by the increase in phone appointments (Table 4).

**Table 4:** Types of advice for face-to-face and phone appointments

	Appointment *n* = 12 224		Phone *n* = 9611	
	No.	%	No.	%
Preventative advice	3393	28	2839	30
Return to work	2123	17	1313	14
Employment/other law	2301	19	2385	25
Disability discrimination	1150	9	1009	10
No advice	2410	20	1076	11
Other^a^	847	7	989	10

Data from pre- and post-COVID groups combined.

a Other includes rehabilitation, other health service, representation at appeal, retirement, vocational guidance.

## Discussion

This study has shown that during the 3 years after the pandemic, there was a slight increase in the number of people with work difficulties associated with psychological problems referred to an occupational health service. This was minor and nothing like the increase expected from recent government statistics (24%).

There was also a slight increase in the number of younger (25–44 years) people referred. During the post-COVID period, there were slightly fewer people referred by GPs, possibly reflecting the fact that advisers were less likely to visit primary care facilities during and after the pandemic. Most consultations are now conducted by phone.

Before putting these findings in the context of current literature, it is important to recognise the strengths and limitations of the current study. The main strength of the study was the stability of the advice centre, which has operated for many years with the same GP practices and advisers working closely together with frequent meetings to discuss the nature of the problems presenting.

There are a number of potential limitations to the study as there were several variables which might have changed during the course of the pandemic, but which could not be measured. These include changes in access to GPs, availability of other sources of advice, and changes in employer-based support. In addition, there was no standardisation of the definitions of presenting health problems. The latter should not have greatly distorted our results, however, as there is no reason why the recording of health problems would change pre- and post-COVID. The small number of advisers who were recording health problems work closely together and regularly discuss cases that they have seen. A full assessment of the mental and physical problems experienced by people attending SOHAS requires externally funded research staff. We plan to do this in the future.

The health problem would usually be a single primary difficulty, such as tennis elbow in a person whose job includes frequent heavy lifting. But some problems are complex with both physical and psychological aspects. An example would be marked back pain, which has led to much time off work. The latter may have caused conflict between employee and employer, leading to a referral to SOHAS for stress and anxiety concerning job retention. Such a patient might be recorded as having either back pain or psychological problem or both but, in this report, only the primary health problem has been included.

The data enabled us to record any differences associated with the major change from face-to-face to phone consultations. This change was associated with an adviser suggesting “return to work” less frequently. Perhaps this advice is more easily offered when the person can be seen to be well enough to return to work.

A further limitation of the study was that there was no measure of the severity of the mental health problem. It has been noted previously that the odds of long-term sickness absence (greater than 3 weeks in any one year) increase in a dose-response fashion with a measure of depressive severity [9].

Comparison of our data with other studies is limited as there are few comparable services which offer advice on how to cope with work difficulties related to illness, as opposed to services which offer treatment. One population-based study of public servants in Japan found that the COVID-19 pandemic did not significantly influence the pattern of long-term sickness absence (LTSA; defined as any sickness lasting 90 days or more); mental disorders were by far the most frequent illness associated with LTSA both before and after the COVID pandemic [10]. Another Japanese study found that the number of non-public workers with LTSA due to mental disorders did not increase during the COVID-19 pandemic [11]. A Finnish study has shown a marked rise in LTSA due to mental illness over recent years, but the rise became most pronounced in the period 2016–2023 with little clear COVID effect [12]. The UK data show a clear increase in LTSA prior to the COVID pandemic [1]. A Canadian study of healthcare workers found that stresses which were so prominent during the pandemic (e.g. fear of COVID-19, personal protective equipment) reduced subsequently but were replaced by other stresses such as frequent incidents of patient anger and stressed colleagues [13].

The predominance of psychological problems among patients seen in the present study mirrors that seen in Japan among applicants for long-term sickness [14]. Two other studies have found that both mental disorders and musculoskeletal disorders were the most common diagnoses leading to long-term sickness absence [15, 16]. It has been estimated that, from the economic point of view, mental health problems have a significantly larger effect than physical health [17]. People off sick with both physical and mental conditions take longer to return than work than those with a single type of illness [18].

A particular problem since the pandemic concerns employees with long COVID or related symptoms; they are at particular risk of adverse work outcomes [19–21]. One study suggested that pre-infection workplace stressors, such as high job demands and poor supervisor support, might contribute to long COVID development [22].

This small project highlights two common aspects of occupational health in relation to the COVID pandemic. Firstly, population studies have shown widespread increases in anxiety and depression associated with the pandemic, but these may not have been severe enough to limit work capacity; in addition, this wave of distress has largely subsided post-pandemic [23, 24]. On the other hand, there has been a clear rise in LTSA in the UK and other countries, but this appears to have started before the pandemic, although it has been exacerbated by it.

Secondly, it is probable that help for work-related mental health problems is too limited and only a proportion of those who need such assistance actually receive it. Even when such help is available, improvement of common mental disorders following an appropriate psychological intervention tends to be poorly correlated with return-to-work [25]. This emphasises the need for services such as SOHAS, which specifically assist people with the interface between health problems and work difficulties.

The local situation, as reported in this study, is one that is largely determined by local referral patterns; the latter do not reflect sickness absence in the local population. In the UK as a whole only 45% of UK workers have access to occupational health services, and this is probably the situation in Sheffield [26]. The present report indicates the importance of a local service for those without ready access to an occupational health service, where appropriate advice is available to patients who have consulted their GP or self-refer with health problems interfering with their work. The referral pathway to SOHAS, mostly through primary care, means that only selected patients with anxiety and depressive disorder are referred to SOHAS. Among employees struggling with health problems that interfere with work, little is known about the decision to seek help from GPs (or self-refer to SOHAS) and how GPs decide which patients to refer to SOHAS. It is likely that there are many people, especially in the younger age groups, who are not working because of mental ill-health but who do not seek help from SOHAS. Future research could usefully determine the factors associated with help-seeking by people struggling with health problems at work, and we need to understand more completely what determines absence from work.

Key learning points **What is already known about this subject:** 

- Psychological problems interfering with ability to work have increased in the UK population since the COVID-19 pandemic.
- It is not known whether more people have sought occupational advice for psychological problems since COVID.

**What this study adds:** 

- There was no marked increase in referrals of people with psychological problems post-COVID to the Sheffield service possibly because the referral pattern does not reflect the prevalence of psychological problems in the local population.

**What impact this may have on practice or policy:** 

- The study showed that the switch from face-to-face appointments to telephone consultations made little difference to the advice offered to patients attending the advisory service; psychological problems formed the most common reason for seeking help.
